# Aortic Thrombus with Bilateral Renal Infarcts: A Case Report

**DOI:** 10.5811/cpcem.7225

**Published:** 2024-01-23

**Authors:** Lev Libet

**Affiliations:** Kern Medical, Department of Emergency Medicine, Bakersfield, California

**Keywords:** thrombophilias, protein C deficiency, protein S deficiency, renal infarction

## Abstract

**Introduction:**

The presence of a hypercoagulable state predisposes to venous and arterial thrombi. While the relationship between protein C and S deficiencies with venous thrombus formation is clear, the relationship to arterial thrombi formation is less common. Thromboembolic disease of the renal arteries may result in renal infarction. The development of simultaneous bilateral renal infarction is rare and can lead to significant morbidity and mortality.

**Case Report:**

This is a case of a 48-year-old male with known protein C deficiency who presented to the emergency department with sudden onset abdominal pain. A computed tomography angiogram of the abdomen showed bilateral renal infarctions. The patient required significant analgesia and developed acute kidney injury. He was treated conservatively, and dialysis was not required.

**Conclusion:**

There are no reports in the emergency medicine literature of bilateral renal infarction secondary to protein C and S deficiency. Prompt evaluation with definitive imaging is necessary for patients who are at high risk for arterial thrombi and present with symptoms suggestive of the diagnosis.

Population Health Research Capsule What do we already know about this clinical entity?

*Renal artery infarction is an uncommon presentation of abdominal pain in the emergency department.*

What makes this presentation of disease reportable?

*A presentation of acute abdominal pain secondary to bilateral renal artery infarction in the setting of protein C and S deficiencies has not been previously reported in the EM literature.*

What is the major learning point?

*Protein C and S deficiencies predispose to arterial thrombi and a sufficient index of suspicion is prudent.*

How might this improve emergency medicine practice?

*Elevate the suspicion for renal artery infarction in those presenting with abdominal pain and a hypercoagulable state.*

## INTRODUCTION

Abdominal pain is a common emergency department (ED) complaint comprising 7–10% of all ED visits. Renal artery infarction is a rare cause of abdominal pain. While the most common cause of renal artery infarction is cardiogenic, 6.6% are due to a hypercoagulable state.^1^ Protein C and S deficiencies are clearly linked with venous thromboembolism with a 5–7 fold increase in risk compared with the general population. Arterial thromboembolic manifestations are less common, affecting 6% of those with protein C deficiency.^2^ Renal infarction carries significant morbidity depending on the severity of associated acute kidney injury. In one case series of 44 patients published in 2004, there was a 30-day mortality of 11.4% associated with renal infarction in patients with atrial fibrilation.^3^ We describe a case of a patient with known protein C deficiency who presented with abdominal pain secondary to bilateral renal infarctions.

## CASE REPORT

A 48-year-old male presented to the ED with sudden onset abdominal pain commencing 45 minutes prior to arrival. The pain was described as sharp, non-radiating, and severe and was poorly localized. He had no shortness of breath or chest pain and denied any fevers. He had a history of protein C deficiency with prior arterial thrombi including a chronic aortic thrombus for which he was prescribed warfarin. The patient’s past medical history was also significant for hypertension, and he was known to be non-compliant with his medications. He previously underwent a right iliofemoral embolectomy, bilateral above knee amputations, and placement of an abdominal infrarenal aortoiliac stent graft. He reported regular use of tobacco, cannabis, and methamphetamines.

On examination he was afebrile, with a heart rate of 98 beats per minute, respiratory rate of 22 breaths per minute, and blood pressure of 189/108 millimeters of mercury. He was in severe distress secondary to his abdominal pain, diaphoretic, and moaning. His abdomen was soft and diffusely tender without guarding or rebound.

**Image 1.**
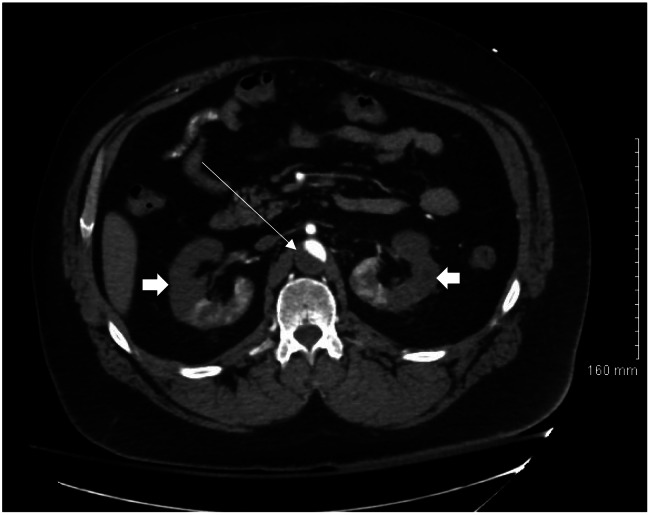
Computed tomography angiogram abdomen in axial plane showing aortic thrombus (thin arrow) and renal cortical hypodensities (thick arrows).

**Image 2.**
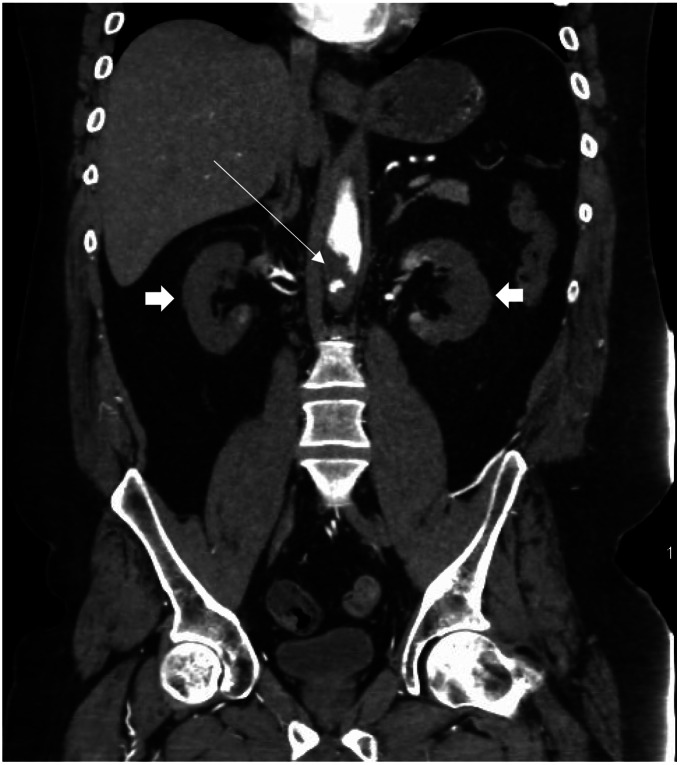
Computed tomography angiogram of abdomen in coronal plane showing aortic thrombus (thin arrow) and hypodense renal cortices (thick arrows).

**Image 3.**
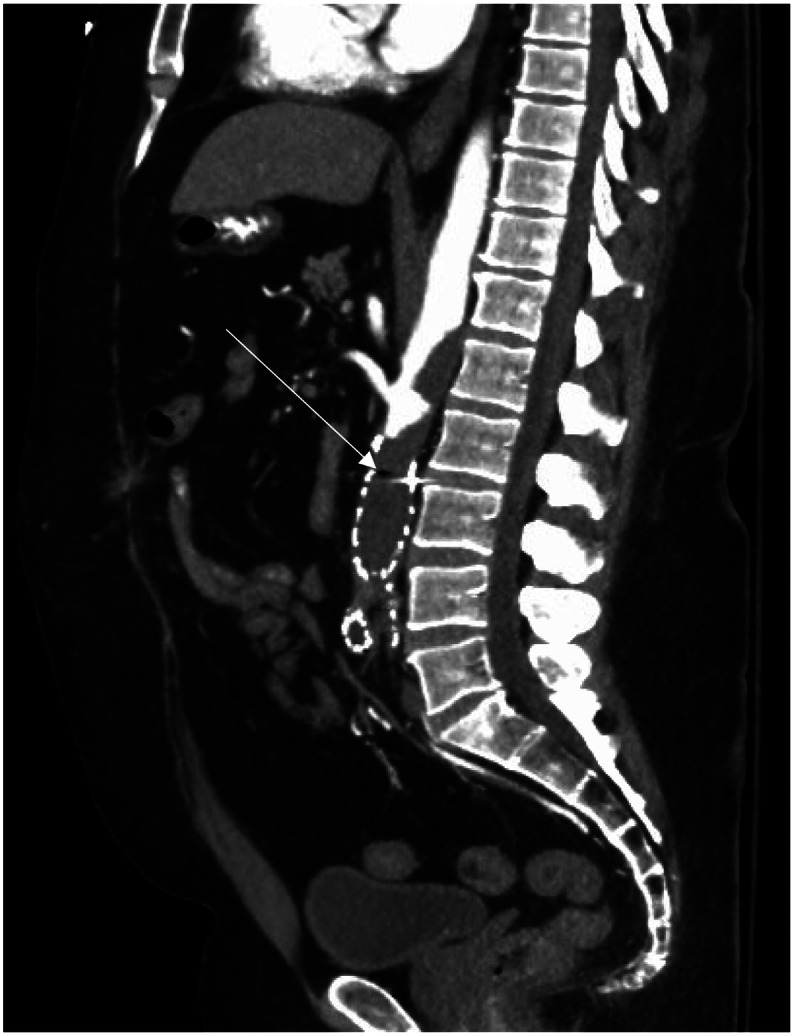
Computed tomography angiogram abdomen in sagittal plane showing degree of clot burden within aortic stent graft (thin arrow).

Laboratory evaluation was remarkable for white blood cell count 23 × 10^3^ per microliter (μL) (reference range: 4.5–11.0 × 10^3^/μL), hemoglobin 15.5 grams per deciliter (g/dL) (13.8–17.2 g/dL), international normalized ratio of 1.02 (<1.1), lactic acid 2.8 millimoles per liter (mmol/L) (0.5–2.2 mmol/L), bicarbonate of 17 milliequivalents (mEq)/dL (22–29 mEq/dL), normal anion gap, and creatinine of 2.13 milligrams (mg)/dL (0.67–1.17 mg/dL) with the patient’s baseline creatinine 1.14 mg/dL. Computed tomography (CT) angiogram of the abdomen and pelvis showed significant thrombus burden from the suprarenal aorta down to the femoral arteries, with near complete occlusion at the level of the renal arteries. Hypodensities of the renal cortices bilaterally indicating bilateral renal infarcts were also noted (Images 1–3).

While in the ED, the patient required several doses of hydromorphone, and a dose of sub-dissociative ketamine. Vascular surgery and interventional radiology were consulted, and both recommended against surgical or endovascular intervention citing questionable benefit with significant risk. Anticoagulation with a heparin infusion was started, and the patient was admitted to the intensive care unit. During the patient’s hospitalization protein S deficiency was also diagnosed. The elevated lactic acid normalized by the following day, and his creatinine peaked at 5.28 mg/dL on day 5 of hospitalization. He did not require dialysis and was discharged on day seven of hospitalization.

## DISCUSSION

Bilateral renal infarction is a rare etiology of abdominal pain. From a sample of all ED visits, the incidence of diagnosed renal infarction is 0.004%.^4^ Of these, the presence of bilateral infarctions is even more uncommon making up only 4.5–20% of renal infarction cases.^3^ Oh et al noted 16.9% had bilateral involvement. Thrombophilias increase the risk of thromboembolism as compared with the general population; however, the highest risk emboli are secondary to cardiogenic sources.^1^ ^,^ ^4^ Most patients with renal infarction present more than 24 hours after the onset, and the majority have generalized abdominal or flank pain.^5^ Many have nausea and vomiting, and 16% have fever.^5^ Our patient presented with severe, abrupt generalized abdominal pain and arrived to the ED within 45 minutes of onset.

Protein C is a vitamin K-dependent proenzyme produced by the liver that activates when bound to thrombin. The activated form is creatively called activated protein C (APC). Protein S is a glycoprotein, also produced in the liver, which acts as a cofactor for protein C. Together APC and protein S participate integrally in the endogenous anticoagulation system mostly via proteolysis of factors V and VIII. Protein C deficiency affects 0.2–0.5% of the population, with clinically significant protein C deficiency being present in only 1 in 20,000 individuals. The association between protein C deficiency and venous thromboembolism (VTE) is well established with a 7-fold increase in risk; however, arterial thromboembolism may be present in only 6% of those with protein C deficiency.^2^ The prevalence of protein S deficiency is less clear in part because the laboratory testing is more difficult to interpret. As with protein C deficiency, protein S deficiency is also associated with VTE. The hazard ratio for development of arterial thrombi is 6.9% and 4.6% for protein C deficiency and protein S deficiency, respectively.^11^

Our patient was at higher risk for complications with his known aortic thrombus and the presence of previously placed endovascular stent coupled with his noncompliance with anticoagulation. The patient’s acute abdominal pain was likely due to new renal infarcts and less likely related to sudden occlusion of the infrarenal aorta and iliac arteries. The presence of aortic thrombus in the setting of protein C deficiency has previously been described.^6^ ^–^ ^8^ Kulahcioglu et al described the only other case we found of bilateral renal infarcts in a patient with protein C deficiency.^9^

While others have noted bilateral renal artery involvement in those with hypercoagulable states in the form of case series, this is the first case to be reported in the emergency medicine literature.^1^ ^,^ ^3^ In our case the patient suffered acute kidney injury but did not require hemodialysis. The incidence of renal dysfunction associated with renal infarcts is between 5–19%, with 2.1–9.1% requiring hemodialysis.^1^ ^,^ ^3^ In the studies summarizing a heterogeneous group with renal infarction, the mortality is between 5–10.2%.^1^ ^,^ ^3^ Further research will more fully describe the risk of renal infarction in the setting of protein C and protein S deficiencies and the resulting clinical effects.

## CONCLUSION

Renal infarction is an important consideration in acute, sudden onset abdominal pain. While arterial thromboembolism is uncommon, the morbidity is significant, and the possibility must be considered in at-risk patients.
